# Sleep Detection for Younger Adults, Healthy Older Adults, and Older Adults Living With Dementia Using Wrist Temperature and Actigraphy: Prototype Testing and Case Study Analysis

**DOI:** 10.2196/26462

**Published:** 2021-06-01

**Authors:** Jing Wei, Jennifer Boger

**Affiliations:** 1 School of Computing and Information System The University of Melbourne Melbourne Australia; 2 Department of Systems Design Engineering University of Waterloo Waterloo, ON Canada; 3 Schlegel Research Chair in Technology for Independent Living Research Institute for Aging Waterloo, ON Canada

**Keywords:** sleep monitoring, wearables, accelerometer, wrist temperature, circadian rhythm, younger adults, older adults, dementia, mobile phone

## Abstract

**Background:**

Sleep is essential for one’s health and quality of life. Wearable technologies that use motion and temperature sensors have made it possible to self-monitor sleep. Although there is a growing body of research on sleep monitoring using wearable devices for healthy young-to-middle-aged adults, few studies have focused on older adults, including those living with dementia.

**Objective:**

This study aims to investigate the impact of age and dementia on sleep detection through movement and wrist temperature.

**Methods:**

A total of 10 younger adults, 10 healthy older adults, and 8 older adults living with dementia (OAWD) were recruited. Each participant wore a Mi Band 2 (accemetry-based sleep detection) and our custom-built wristband (actigraphy and wrist temperature) 24 hours a day for 2 weeks and was asked to keep a daily sleep journal. Sleep parameters detected by the Mi Band 2 were compared with sleep journals, and visual analysis of actigraphy and temperature data was performed.

**Results:**

The absolute differences in sleep onset and offset between the sleep journals and Mi Band 2 were 39 (SD 51) minutes and 31 (SD 52) minutes for younger adults, 49 (SD 58) minutes and 33 (SD 58) minutes for older adults, and 253 (SD 104) minutes and 161 (SD 94) minutes for OAWD. The Mi Band 2 was unable to accurately detect sleep in 3 healthy older adults and all OAWDs. The average sleep and wake temperature difference of OAWD (1.26 °C, SD 0.82 °C) was significantly lower than that of healthy older adults (2.04 °C, SD 0.70 °C) and healthy younger adults (2.48 °C, SD 0.88 °C). Actigraphy data showed that older adults had more movement during sleep compared with younger adults and that this trend appears to increase for those with dementia.

**Conclusions:**

The Mi Band 2 did not accurately detect sleep in older adults who had greater levels of nighttime movement. As more nighttime movement appears to be a phenomenon that increases in prevalence with age and even more so with dementia, further research needs to be conducted with a larger sample size and greater diversity of commercially available wearable devices to explore these trends more conclusively. All participants, including older adults and OAWD, had a distinct sleep and wake wrist temperature contrast, which suggests that wrist temperature could be leveraged to create more robust and broadly applicable sleep detection algorithms.

## Introduction

On average, we spend one-third of our lives asleep [1]. Quality and quantity of sleep regulate daytime behaviors and functions as well as significantly impact health and well-being. Sleep has been shown to affect day-to-day memory and concentration [2-4]. Lifetime sleep habits appear to correlate with one’s likelihood of having conditions, such as Alzheimer disease [5-7] and cardiovascular diseases [8,9]. Although the benefits of getting a good night’s rest have received extensive research, there is still much we do not know about the differences between how people sleep and what can be done to support better sleep.

Understanding sleep patterns is a complex undertaking, as sleep is impacted by conscious and subconscious control by the individual as well as environmental factors. Standard clinical sleep evaluation typically uses polysomnography (PSG), which is considered the *gold standard* in sleep studies [10]. PSG requires the person being assessed to sleep in a laboratory with devices attached to the body in single or infrequent sessions. Although the data collected by PSG can be used to diagnose many medical conditions, its intrusive, unnatural nature might not reflect people’s usual sleep quality and is not appropriate for long-term or frequent monitoring of sleep [11].

**Figure 1:**
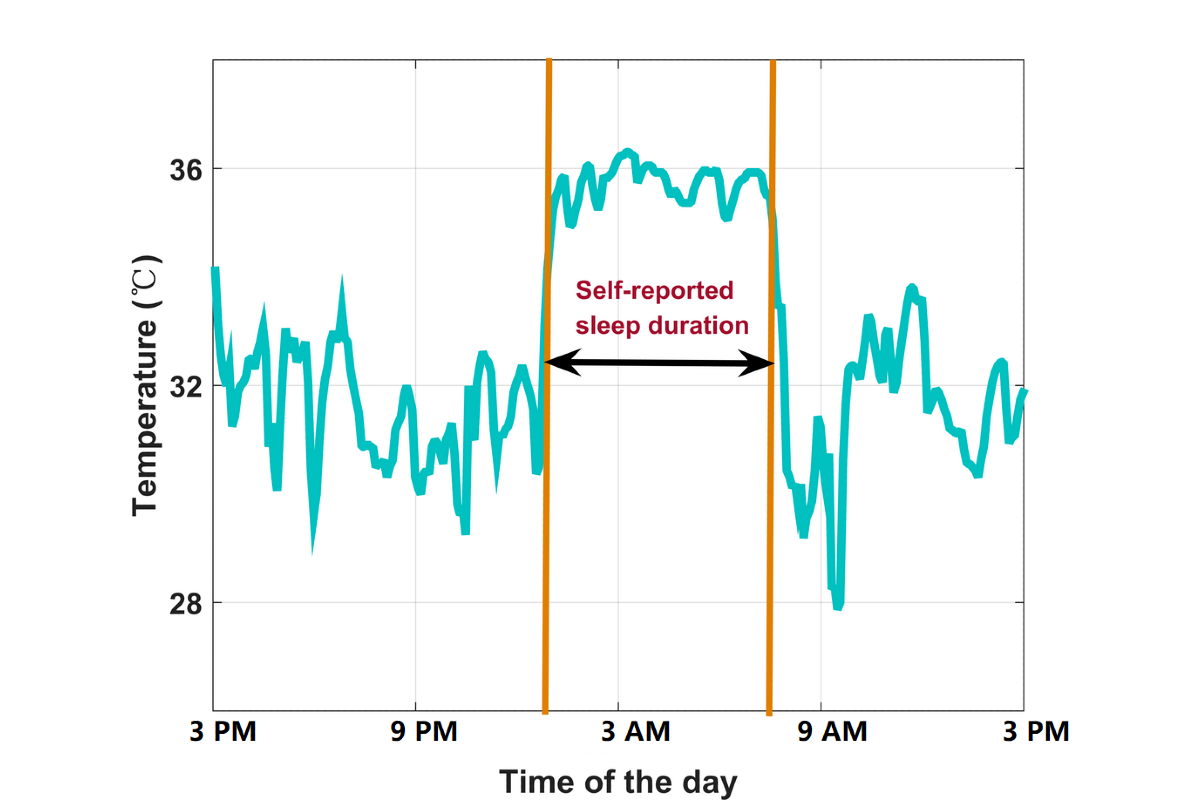
Example wrist temperature over a 24-hour period for a healthy sleep pattern.

Actigraphy (ie, accelerometer sensors and gyroscopes) is being increasingly used to measure people’s sleep and activities by estimating related parameters, such as sleep onset and offset [12,13]. Many clinical studies have adopted actigraphy to measure circadian rhythm, which is a major factor in regulating people’s sleep and wake rhythms [14,15]. Driven by circadian rhythms, many biological processes, including core body temperature (CBT), have 24-hour diurnal variations. During sleep, people’s CBT drops about 1 °C [16]. Thermal regulation inside the human body causes wrist temperature to exhibit an opposite pattern to CBT [17]; wrist temperature increases before people fall asleep, remains at a relatively high level when people are asleep, and then drops in the morning when people wake up (Figure 1). CBT has been used extensively to study circadian rhythms; however, it usually requires invasive gut or rectal temperature measurements [18]. As an alternative, wrist temperature can be measured in daily life and is found to be more correlated with sleeping status than CBT [19]. Recent studies have provided increasing evidence that wrist temperature can be used for sleep monitoring [17,20-22].

Low sleep quality and irregular sleep patterns become more prevalent with increasing age and dementia [23-26]. For example, poor nighttime sleep has been shown to be a key factor in older adults’ tendency to have more daytime sleepiness [27], which has been associated with changes in circadian rhythms in older adults [28]. Sleep can also be used as an indicator of comorbidities, such as sleep apnea and restless leg syndrome, both of which become more prevalent with increasing age [29,30]. Disrupted sleep patterns have been found to be even more prevalent in people with dementia [31]. Poor sleep has been found to be predictive of more severely impacted cognition in older adults, including people with dementia [32] and older women without dementia [33]. Therefore, the ability to monitor older adults’ sleep and circadian rhythms on an ongoing basis is increasingly being used to understand and support their sleep, which, in turn, supports health and well-being [34].

The proliferation of smart wearable technologies has contributed to the substantial growth in technology adoption among older adults; older adults are the most rapidly increasing group of users of new technologies [35,36]. One of the most commonly adopted technologies is smart wearable devices, which usually have built-in accelerometer and gyroscope sensors to detect people’s sleep (eg, Fitbit, Garmin, and Mi Band) [37-39]. Commercial smart wristbands have been designed to output several sleep parameters, including sleep onset, offset, duration, and wake after sleep onset, and some wristbands also give a sleep quality score. Sleep parameters can be synchronized to one’s smartphone via Bluetooth (and then to the cloud, should they wish) so that users can easily access their sleep data. Relative affordability and unobtrusiveness contribute to the growing popularity of commercial smart wristbands. In addition, smart wearable devices are being increasingly and widely used in health research [40-42]. For example, Gibson et al [43] evaluated the reliability of actigraphy to measure the sleep of people living with dementia.

Although most smart wristbands can provide sleep monitoring and analysis, they fail to monitor people’s sleep from an internal perspective; they cannot measure circadian rhythm directly. As discussed earlier, wrist temperature is a reflection of CBT, which is indicative of a person’s biological circadian rhythm. As such, wrist temperature could have value as an addition to accemetry-based wearable sleep monitoring systems, which could then be used to support better understanding and management of sleep. To be successful, these systems should work for different stakeholder groups, including older adults and older adults living with dementia (OAWD). Therefore, this research was guided by the question, “How do sleep and wrist temperature patterns compare for younger adults and older adults, including OAWD?”

## Methods

### Participants

This study measured sleep patterns and wrist temperature patterns of 3 groups of participants: (1) healthy younger adults (aged 20-30 years), (2) healthy older adults (aged≥65 years), and (3) OAWD. Inclusion criteria were any gender, any race, age in the targeted populations, and ability to understand English. An additional criterion for OAWD was early- to middle-stage dementia. Individuals diagnosed with severe sleep disorders were excluded from the study.

We recruited 10 participants for each group based on the convenience sampling method. Healthy adults were recruited through university-wide poster advertisements and word of mouth. Healthy older adults were recruited through phone calls and email invitations. OAWD participants were recruited from a local long-term care (LTC) residence. The staff at the LTC setting requested that they coordinate the initial identification of potential participants and support the recruitment process. Each potential participant was then contacted and introduced to the study by the researcher. Assent to continue participating in the study was obtained from each OAWD participant every time they were contacted by the researchers. This study was approved by the Human Research Ethics Committee of the University of Waterloo (ORE #31860 and ORE #40459).

### Equipment

**Figure 2:**
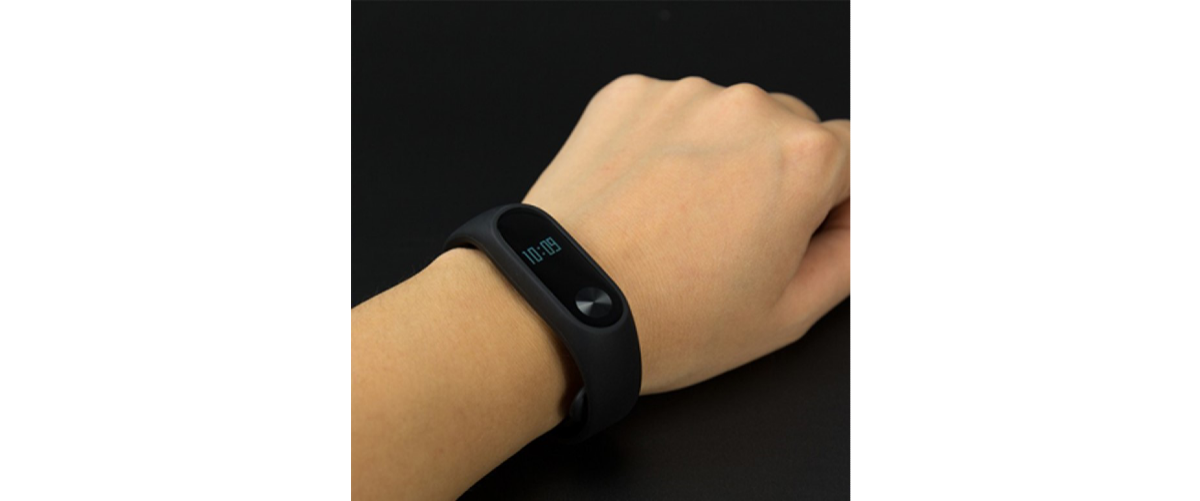
The Mi Band 2.

**Figure 3:**
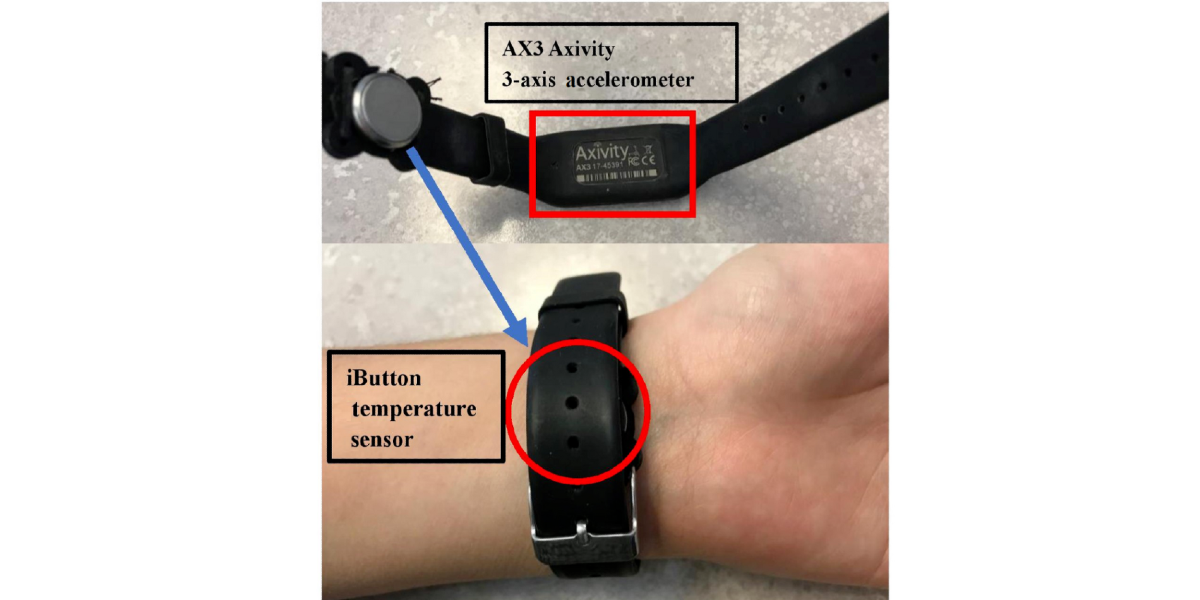
The customized wristband.

Each participant was asked to wear a commercially available smart wristband (Mi Band 2, XiaoMi; Figure 2) and a custom-built wristband developed by the authors (Figure 3) on their left arm for 14 days. The Mi Band 2 was chosen because of its popularity, low cost, long battery life, and accessibility of actigraphy data. The custom-built band consisted of a temperature sensor (iButton DS1922L, Maxim) that maintained contact with the anterior side of the wrist and a 3-axis accelerometer sensor (AX3, Axivity). Both sensors operated offline by storing data locally; data were extracted after the 2-week study period using USB adaptors. All participants were offered to keep the Mi Band 2 if they wished on completion of the study.

### Study Protocol

The study protocol was the same for healthy younger adults and healthy older adults, with an altered OAWD protocol. Before the start of the study, all healthy participants were asked to complete 4 questionnaires: (1) a demographic form; (2) morningness-eveningness questionnaire, which is a measure of *circadian rhythm type* [44]; (3) Pittsburgh Sleep Quality Index (PSQI), which is a measure of subjective sleep quality [45]; and (4) Epworth Sleepiness Scale (ESS), which is a measure of daytime sleepiness [46]. Participants were then given the 2 wristbands; instructed on how to wear them; and asked to wear both on their left wrist for 14 days, except when showering or bathing. Participants were also asked to fill out a daily sleep journal that was adapted from a study by Monk et al [47] to capture sleep onset, offset, and subjective sleep quality.

As OAWD were not able to fill out the questionnaires, only the demographic form was completed and was done with the help of staff from their LTC residence. In addition, the Mini-Mental State Examination (MMSE) [48] was conducted to approximate the level of cognition for each OAWD. After giving each OAWD the wristbands, their personal support workers (PSWs) were asked to help with getting wristbands off and on when they assisted OAWD shower or bathe as well as to routinely check that the person was not adversely affected by wearing the wristbands. In addition, PSWs were asked to observe participants’ sleep status and check off either *awake* or *sleeping* on a sheet every half an hour throughout the day and night as part of their routine in with the OAWD. Sleep status from this sheet was then used to generate an observed sleep journal.

### Ground Truth of Sleep Parameters

Sleep onset and offset, the number of nighttime wake-ups, and sleep quality scores were obtained from the sleep journals and were used to help interpret actigraphy and temperature data. Although we thought it was unlikely that people could estimate their sleep at a minute level, we considered sleep journals of younger adults and healthy older adults reliable at the half-hour interval level. Occasionally, participants would miss reporting sleep onset and offset. In these cases, the sleep onset and offset were manually identified from the data from the custom-built wristband AX3 actigraphy data.

For OAWD, the intention was to use the sleep status sheet filled out by PSWs to extract sleep parameters and use these as ground truths for sleep. However, at the end of the study, it was found that, on average, 40% of the sheet was filled (ie, the PSWs did not report sleep status of OAWD 60% of the time). Owing to the large amount of data missing from the OAWD sleep journals, we chose to manually identify sleep periods from the AX3 data and combined those identified sleep periods with PSW sheets to estimate sleep for all OAWD.

### Feature Extractions

#### Sleep Parameter Extraction

Sleep onset, sleep offset, and sleep duration from the sleep journals were compared with the Mi Band 2 for daily sleep monitoring; the mean values across the 14 days were calculated and are presented in Table 1. The absolute differences between the Mi Band 2 and journal parameters were used to indicate the agreement of sleep detection between Mi Band 2 and self-reported sleep.

**Table 1:** Sleep parameters calculated from sleep journals (younger and older adults) and recreated sleep journals (older adults living with dementia).

Sleep parameters	Younger adults	Older adults	Older adults living with dementia
Onset, mean (SD)	12:32 AM (53 min)	11:49 PM (61 min)	9:37 PM (105 min)
Offset, mean (SD)	7:55 AM (54 min)	7:31 AM (78 min)	7:05 AM (40 min)
Duration (min), mean (SD)	442 (26)	462 (88)	562 (123)

#### Sleep Periods Identification of OAWD

As people generally have minimal movements during sleep, sleep periods can be manually identified by segments of raw AX3 data that have minimal variations. For one night’s sleep, multiple sleep periods could be identified if the person had multiple wake-ups. Once sleep periods were estimated, the sleep onset, offset, and duration were calculated for the longest least varied data period. If participants woke up during the night and remained awake for a long period (ie, more than 2 hours), this period was not counted as sleep duration. AX3-estimated sleep was combined with the partially completed observational sheet by PSWs to create a recreated sleep journal for OAWD.

#### Wrist Temperature Feature Extraction

As participants took the customized wristband off to shower, all data points lower than 28 °C were removed and interpolated using the nearest values. The temperature data were then smoothed by applying a median filter with a window size of 3. Descriptive statistics were calculated to provide a profile of the general characteristics of the participants’ wrist temperature patterns and rhythm indices related to circadian rhythms. The mean and SD were calculated for the wrist temperature rhythm of every 24-hour period that began at 3 PM because this segmentation included full nighttime sleep periods and daytime naps. On the basis of sleep onset and sleep offsets obtained from sleep journals, the mean temperatures were calculated for sleep time and wake time. The mean wake time temperature was subtracted from the mean sleep time temperature to calculate the mean sleep and wake temperature difference, which was used as a measure of the extent of temperature changes between the sleep and wake states in this study.

Interday stability (IS) and intraday variability (IV) were calculated to examine the regularity and rhythmicity of wrist temperature rhythms for each participant [49]. IS has been used to measure the stability of rhythms across several consecutive days, whereas IV reflects the fragmentation of the wrist temperature rhythm of every 24-hour period. IS and IV were calculated using Equations (1) and (2), respectively:

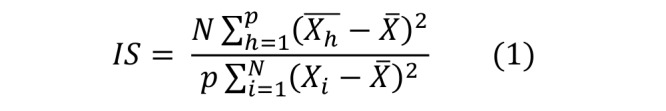

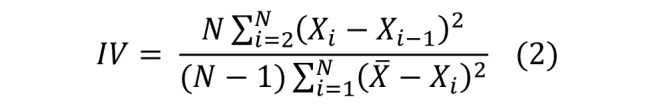





where *N* is the total number of wrist temperature data points, *p* is the number of wrist temperature data points per day (in this study, *P*=288 as the iButton is sampled every 5 min), *X* is the mean of all temperature data, *X_h_* is the hourly mean of wrist temperature, and *X*_i_ is the data point at time *i*. IS and IV were calculated for each participant for the total study period.

### Statistical Analysis

One-tailed *t* test was used to compare the sleep parameters and rhythm indices between different groups (young vs old and old vs OAWD). The Pearson correlation coefficient was used to assess correlations between different circadian rhythms and sleep parameters calculated; *P*<.05 was considered to be significant.

### Case Study Analysis

When analyzing the collected data, it was found that the sleep of some participants was not correctly reported by the Mi Band 2. For example, most PSWs reported that OAWD had 8 hours of sleep at night, but the Mi Band 2 detected as few as 40 minutes of sleep. The AX3 data from the custom-built band were inspected for the periods Mi Band 2 had classified as *wake periods* during the night as well as wrist temperature measurements for the same time. A healthy younger participant who had good sleep quality; a healthy older participant with poor sleep quality; and 2 OAWD who had poor, interrupted sleep were selected as case studies to illustrate this phenomenon and are presented later in this paper.

## Results

### Demographics and Sleep Patterns

In total, we recruited 10 healthy younger adults, 10 healthy older adults, and 8 OAWD. A total of 7 OAWD were able to give their own consent and sign the consent form; for the 1 participant who could not self-consent, his power of attorney signed the consent form, and assent was obtained from the participant. Participants’ demographic data are presented in Table 2. The average MMSE score of the 8 OAWD participants was 20. Moreover, 7 OAWD were in the mild cognitive impairment category (ie, a score between 18 and 23), and 1 OAWD had a score of 16, which indicates severe cognitive impairment [50].

**Table 2:** Participants’ demographics.

Demographic		Healthy younger adult group (n=10)	Healthy older adult group (n=10)	Older adults living with dementia group (n=8)
Age (years), mean (SD)		24.1 (2.23)	75 (7.36)	83.25 (10.38)
**Sex, n (%)**				
	Male	4 (40)	4 (40)	4 (50)
	Female	6 (60)	6 (60)	4 (50)
BMI (kg/m^2^), mean (SD)		23.69 (6.30)	25.94 (3.11)	29.90 (8.91)
Pittsburgh Sleep Quality Index, mean (SD)^a^		5.2 (2.39)	7.8 (3.56)	—^b^
Mini-Mental State Examination, mean (SD)^c^		—	—	20 (2.39)

^a^The Pittsburgh Sleep Quality Index was not measured for the older adults living with dementia group as they cannot reliably complete this measure.

^b^Test was not administered as the test was not appropriate for the participant group.

^c^The Mini-Mental State Examination of the healthy younger adult group and the healthy older adult group as they were cognitively intact.

The sleep parameters obtained from the sleep journals for all participants are presented in Table 1. Compared with older adult groups, healthy younger participants tended to sleep later and wake up later, with the shortest average sleep duration among the 3 groups. Between the 2 older adult groups, OAWD slept earlier and had a longer sleep duration. Although we did not require participants to record daytime naps, we asked them to report whether they had the habit of napping. Only 1 younger adult and 1 healthy older adult reported that they took regular noontime naps. All OAWD napped during the day, sometimes more than once. As the recreated sleep journals of OAWD only summarized nighttime sleep and did not capture daytime naps, the total sleep duration of OAWD might be longer than reported. Healthy older adults had better compliance in reporting and provided more detailed sleep journals (eg, a few participants consistently recorded their nap time during the day).

### Comparison Between Mi Band 2 and Sleep Journal

The absolute differences in sleep parameters between the Mi Band 2 and sleep journals are summarized in Table 3, and significant differences were found in sleep duration between the younger adult group and the healthy older adult group. Between the 2 older adult groups, the absolute differences in sleep onset, offset, and duration of OAWD were significantly higher than those in the healthy older adult group. As shown in Table 4, of the 106 days of data measured from the 8 OAWD, for 28 of the days (28/106, 26% of days monitored), the Mi Band 2 did not detect any sleep, and we confirmed that all OAWDs were wearing the Mi Band 2 correctly throughout the study. Discrepancies between sleep parameters reported in the recreated sleep journal and detected by Mi Band 2 for OAWD were large, namely, sleep onsets detected by Mi Band 2 were often noticeably delayed (ie, on the order of hours), and the offset was earlier than what was reported. The average absolute difference in sleep duration between the Mi Band 2 data and the recreated sleep journal for OAWD was more than 6 hours. A discussion of this detection error is presented later in the *Case Studies* section.

**Table 3:** The absolute difference of sleep parameters between (recreated) sleep journals and the Mi Band 2 for each group.

Sleep parameters		Healthy younger adults	Healthy older adults	Older adults living with dementia
**Onset**				
	Difference (min), mean (SD)	39 (51)	49 (58)	253 (104)
	*P* value	—^a^	—	<.001
**Offset**				
	Difference (min), mean (SD)	31 (52)	33 (58)	161 (94)
	*P* value	—	—	<.001
**Duration**				
	Difference (min), mean (SD)	49 (59)	64 (77)	379 (163)
	*P* value	—	.04	<.001

^a^No significance.

**Table 4:** Number of days with sleep reported by the Mi Band 2 for older adults living with dementia.

ID	Total days (n)	Valid days, n (%)
OAWD^a^ 1	14	14 (100)
OAWD 2	13	2 (15)
OAWD 3	10	10 (100)
OAWD 4	14	7 (50)
OAWD 5	14	12 (86)
OAWD 6	13	8 (62)
OAWD 7	14	11 (79)
OAWD 8	14	14 (100)
Total	106	78 (74)

^a^OAWD: older adults living with dementia.

### Wrist Temperature Rhythm Comparison

**Figure 4:**
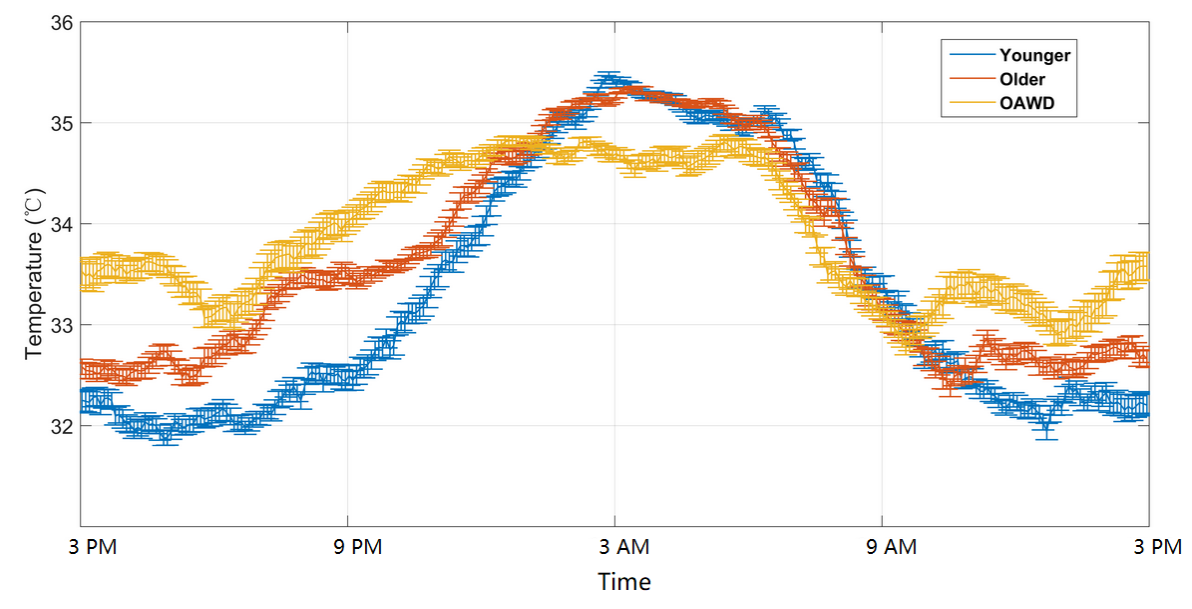
Average wrist temperature patterns for healthy younger adults, healthy older adults, and older adults living with dementia. Plots are expressed as the mean temperature (standard errors of the mean). OAWD: older adults living with dementia.

The average wrist temperature data of the 3 groups are shown in Figure 4. Although all curves have higher daytime (awake) temperatures, the OAWD group has the flattest temperature curve with the lowest nighttime wrist temperature and the highest daytime wrist temperature compared with the other groups. Although the average nighttime wrist temperature was similar for healthy older and younger adults, older adults tended to have a higher daytime wrist temperature, causing a flatter average curve for healthy older adults compared with younger adults. The wrist temperature indices are presented in Table 5.

**Table 5:** Wrist temperature indices.

Parameters			Healthy younger adults (n=10)		Healthy older adults (n=10)		Older adults living with dementia (n=8)
**Average wrist temperature parameters (°C), mean (SD)**							
	Sleep temperature	34.98 (0.55)		34.97 (0.34)		34.66 (0.53)	
	Wake temperature	32.49 (0.83)		32.94 (0.67)		33.40 (1.03)	
	Sleep and wake temperature difference	2.48 (0.88)		2.04 (0.70)		1.26 (0.82; *P*=.02)	
**Cosinor analysis**							
	MESOR^a^ (°C), mean (SD)	33.34 (0.61)		33.65 (0.46)		33.90 (0.84)	
	Amplitude (°C), mean (SD)	1.72 (0.57)		1.45 (0.50)		0.93 (0.59; *P*=.03)	
	Acrophase (min), mean (SD)	246 (59)		183 (91; *P*=.04)		44 (145; *P*<.001)	
**Nonparametric analyses (°C), mean (SD)**							
	Interday stability	0.53 (0.10)		0.52 (0.16)		0.32 (0. 19; *P*=.02)	

^a^MESOR: midline-estimating statistic of rhythm.

### Case Studies

Data from 4 participants are presented below as illustrative case studies. One of the case studies is of a younger *healthy sleeper*, and the other 3 cases are representative data from *irregular sleepers*, 1 of which is a healthy older adult and the other 2 are OAWD.

#### Case I: Healthy Younger Adult With Regular Sleep

**Figure 5:**
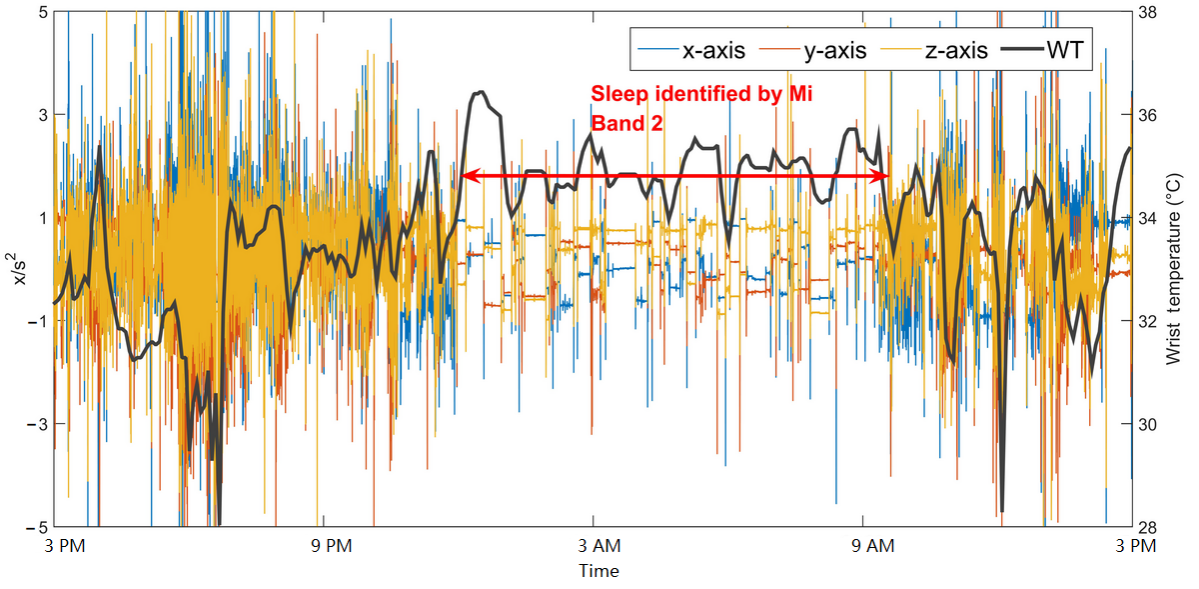
Example wrist temperature and AX3 data for a healthy younger adult with good sleep (YA1). Sleep onset and offset detected by Mi Band 2 are indicated by the double red arrows. WT: wrist temperature.

The healthy younger adult (YA1) was a 25-year-old man (PSQI=5 and ESS=3) who did not report any sleep disorders. One day of YA1 data, including wrist temperature, AX3 data, and sleep onset or offset detected by Mi Band 2, is shown in Figure 5. The sleep period and wake period can be distinguished using AX3 data, which shows that YA1 was mostly static with a relatively high and stable wrist temperature during sleep. The sleep onset and offset detected by the Mi Band 2 align well with changes in AX3 and wrist temperature data.

#### Case II: Healthy Older Adult With Irregular Sleep

**Figure 6:**
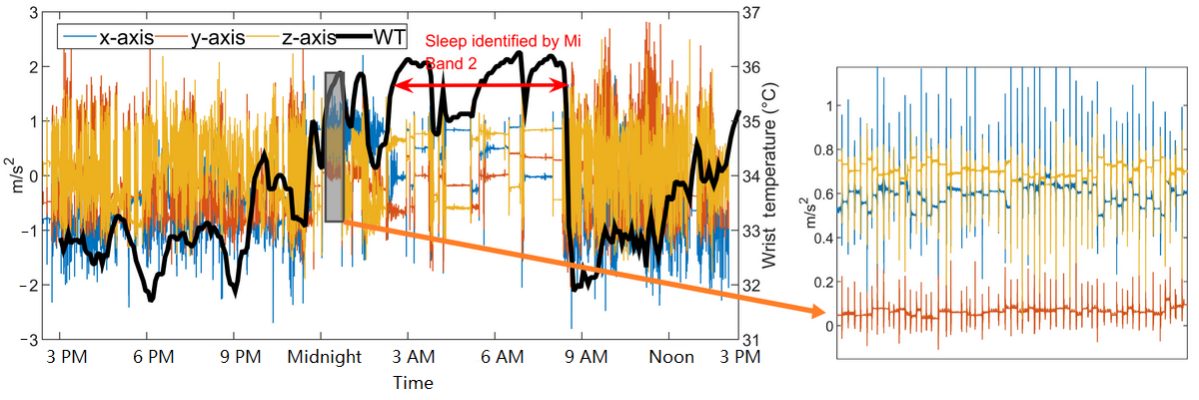
Example wrist temperature and AX3 data for a healthy older adult with irregular sleep (OA1). Sleep detected by the Mi Band 2 is indicated by the red double arrow. WT: wrist temperature.

The healthy older adult (OA1) was an 83-year-old man (PSQI=6 and ESS=7) who did not report any sleep disorders. OA1 was considered to be an *irregular sleeper* based on his data; one day of representative data is shown in Figure 6. Sleep onsets detected by the Mi Band 2 were, on average, 2 hours later than OA1’s self-reported sleep onset.

For the day represented in Figure 6, OA1 self-reported falling asleep at around 11:45 PM, whereas the Mi Band 2 reported that his sleep started at 2:43 AM. Significant movements were captured by AX3 between 12:00 AM and 2:30 AM; a magnified image, except for these data, is shown on the right side of Figure 6, where multiple peaks can be observed in all 3 axes. After 2:30 AM, the AX3 data became more static and similar to the AX3 data collected from *regular sleepers*. The movements between 12:00 AM and 2:30 AM were very periodic and occurred approximately every 30 seconds for most of the 2.5-hour time frame; during this time, the wrist temperature remained relatively high, and the participant reported being asleep. As it seems unlikely that these movements were made by the participant consciously and people cannot *fake* body temperature changes, the participant was likely asleep but experiencing irregular body movements.

#### Case III: OAWD1 With Parkinson Disease and Sleep Apnea

**Figure 7:**
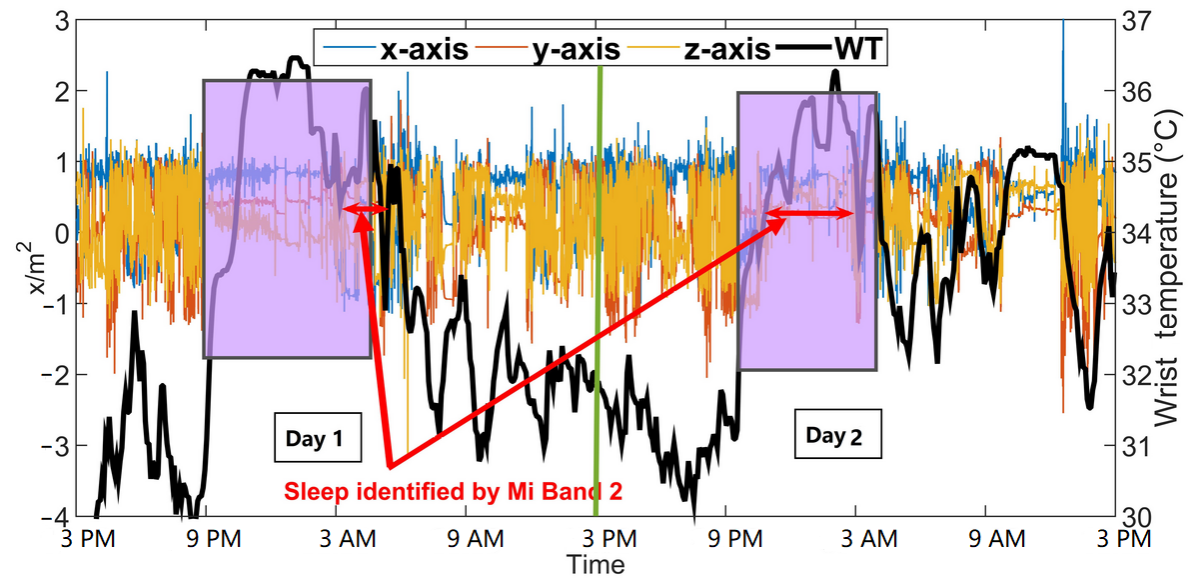
Two consecutive days of wrist temperature and AX3 data for older adults living with dementia 1. Sleep onset or offset were obtained from the recreated sleep journal. Sleep detected by the Mi Band 2 is indicated by the red double arrow. The sleep of 2 nights in the recreated sleep journal is labeled by the shaded boxes. WT: wrist temperature.

OAWD1 was a 68-year-old man (MMSE score=21, diagnosed with Parkinson disease and sleep apnea); 2 days of data are shown in Figure 7. Compared with OA1 (Figure 6), OAWD1’s data (both wrist temperature and AX3 data) have a more evident contrast between day and night. Even so, only parts of his sleep were detected by Mi Band 2. As shown in the left portion of Figure 7, there was little movement between 9:00 PM and 3:00 AM, and the wrist temperature was relatively high, but this time frame was not identified as sleep by Mi Band 2.

#### Case IV: OAWD2 With Sleep Apnea and Insomnia

**Figure 8:**
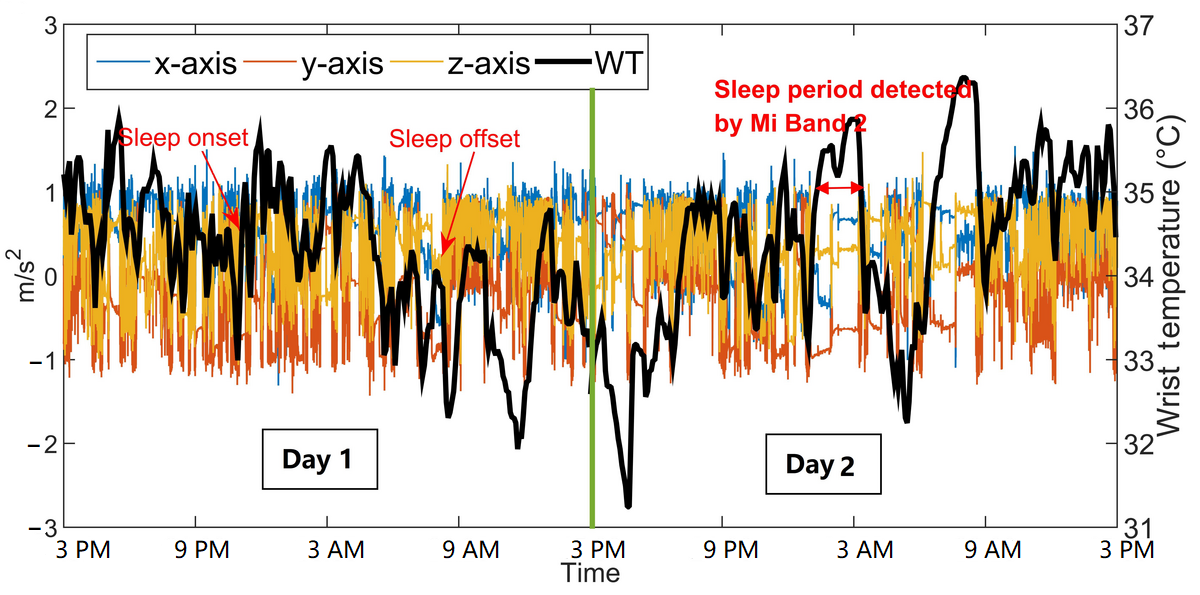
Two consecutive days of wrist temperature and AX3 data of older adults living with dementia 2. Sleep onset or offset were obtained from the recreated sleep journal. WT: wrist temperature.

OAWD2 was a 68-year-old woman (MMSE score=19) who was diagnosed with sleep apnea and insomnia and was an *irregular sleeper*. Before the start of the study, OAWD2’s PSW reported that she had very poor sleep (ie, would wake up at night frequently and sometimes cannot fall asleep at all). During her 13 days in our study, the Mi Band 2 indicated no sleep for 11 days; PSWs confirmed that she wore the wristbands the entire time. Figure 8 shows 2 consecutive days of wrist temperature and AX3 data. The left portion of Figure 8 shows data where no sleep was detected by Mi Band 2, and the right portion shows a short period of sleep detected by Mi Band 2. Compared with data from OA1 in Figure 6, there are no obvious *static periods*, and the day and night contrast for movement is relatively indistinguishable. Although there are differences in daytime and nighttime wrist temperatures, these are more difficult to distinguish than in other participants.

**Figure 9:**
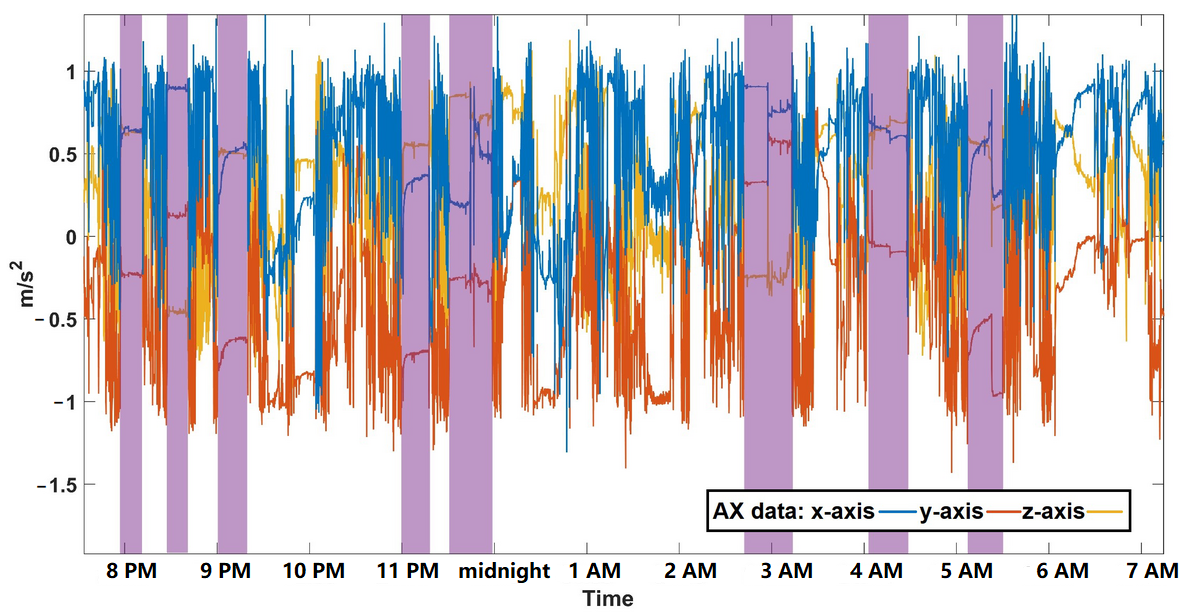
An 11-hour period (between 8 PM and 7 AM) of AX3 data for older adults living with dementia 2. Periods of relatively static data are shown by purple rectangles. WT: wrist temperature.

A close-up of an excerpt of AX3 data for day 1 is shown in Figure 9, where 8 short episodes of static data are highlighted. Each highlighted episode lasted <30 minutes, and considerable movement was observed between episodes. These data indicate that the participant may have had some sleep, but if so, the sleep was in short, fragmented periods. As the participant was diagnosed with sleep apnea, this could partially account for frequent nighttime wake-ups.

## Discussion

### Principal Findings

In line with other recent studies [17,19], this study found that wrist temperature increased when people were asleep, even when their sleep patterns were irregular. As illustrated in the case studies presented earlier, wrist temperature appears to correspond with sleep status changes regardless of whether body movements occurred; wrist temperature increased when people were asleep and decreased when they woke up. This adds evidence that wrist temperature is correlated with circadian rhythms and associated changes in thermal regulation of the body during sleep. This appears to hold true for OAWD as well, whose circadian rhythms are impacted by both aging and dementia (and often other morbidities); their wrist temperature appears to reflect when they are asleep and awake. A larger sample size is needed to draw more definitive conclusions regarding the impact dementia may have on CBT and wrist temperature.

From the results presented in Table 5 and Figure 4, there seems to be a trend of increasing wake temperature and decreasing sleep and wake temperature difference as people age and people become more pronounced dementia in this study. It is not clear from our data whether and how much this increased discrepancy is due to dementia or other factors, such as increased average age of OAWD, greater prevalence of comorbidities, and/or how living in an (often busy) LTC environment may disrupt sleep. The less significant day versus night wrist temperature contrast suggests changes in the circadian rhythms of older adults and OAWD compared with younger adults. This finding is consistent with other studies (eg, [51]).

The IS values suggest that the 2 healthy groups had similar IS for their wrist temperature rhythm, and the dementia group had lower wrist temperature rhythm stability. The lower IV values of the older adult groups indicate that changes in wrist temperature rhythms tend to be less variable intraday as people get older, especially with people who have dementia. As IS reflects the stability and the IV reflects the fragmentation of one’s wrist temperature rhythm, the significantly lower IS and IV of OAWD could be associated with irregular sleep patterns and increased daytime sleepiness. Most OAWD in this study took frequent naps and experienced insomnia at night, which almost certainly increased the average daytime wrist temperature, lowered the average nighttime wrist temperature, and accounted for a greater amount of flux in nighttime wrist temperature. Rhythm indices, such as IS and IV, could be further explored with these and other populations to determine how they might be used to infer circadian rhythm health.

Some studies [30,52-54] have reported excessive body or wrist movements of OAWD during sleep. This research supports the hypothesis that advanced sleep monitoring systems should detect nighttime body movements. Although the exact cause of abnormal body movements by the older adults in this study is unknown, body movement during sleep seems to become increasingly common with age and was present in all our OAWD participants. A system that can detect involuntary movements could be valuable in the detection of previously unknown conditions (such as the case with our OA1 participant) and possibly support the diagnosis and management of conditions that impact sleep.

There are 2 noteworthy findings regarding sleep detection using the Mi Band 2. First, by comparing the results of the Mi Band 2 with AX3 data from the custom-built wristband and reported sleep from sleep journals, it appears that the Mi Band 2 does not accurately detect people’s sleep when they have pronounced or irregular body movements during sleep. Second, the Mi Band 2 sleep detection algorithm does not appear to work well if the user has fragmented sleep (ie, waking up multiple times during the night). For example, OAWD2 slept in short and sporadic episodes; most of her sleep was undetected by Mi Band 2, which reported sleep for only 2 of her 13 recorded days. From the example data in Figure 9, we can observe that each sleep episode of OAWD2 was short and each wake-up accompanied body movements and that the Mi Band 2 did not detect any sleep. As the Mi Band 2 detects sleep based on accemetry data, it is plausible that periodic body movements during sleep, such as those seen in the older adults presented in the case studies, may be the cause of the misclassification of these periods as *awake* instead of *asleep*. Moreover, the Mi Band 2 may be trained on data from people with 1 or 2 long nighttime sleeping periods than multiple short ones. The hypothesis that movements and short sleeping episodes cause the Mi Band to miss episodes of sleep is supported by the data from our other participants.

Although the sleep detection algorithms for most commercial smart wristbands are proprietary information, as they rely on accelerometers and gyroscopes, their sleep detection is based on movement. For a regular sleeper, frequent and substantial body movements at night usually indicate the person is awake, and fewer and infrequent movements indicate sleep. Although an activity-based rule for sleep detection may be robust for people who are mostly still when they sleep, this study shows that sleep detection based on activity is not accurate for some older adults, especially OAWD. As younger adults are the primary consumers of wearable wristbands, the Mi Band 2 sleep detection algorithms may have been trained predominantly on data from younger adults with healthy sleep patterns, thus biasing the algorithm to detect sleep for people who fit that profile. This aligns with other studies that have found that commercial wristbands performed poorly on populations with sleep disorders [55]. Therefore, other smart wristbands that track sleep based on movement alone may have similar performance as the Mi Band 2 for the same reasons; however, this was not investigated in this study; therefore, it remains a question for future research.

This research highlights the need for the older adult population to be included in the development of sleep detection algorithms, including the possibility of developing smarter algorithms that autonomously identify the type of sleeper a person is and self-adjust appropriately to that person’s sleep habits. This requires research into how to categorize what *good* sleep is for that particular person in a way that helps people, their care partners, and/or caregivers understand. This information could then be used to better manage sleep without missing information that may indicate a change in sleep or sleep patterns that are of possible concern. As wrist temperature appears to be highly correlated with sleep regardless of age or dementia, the inclusion of wrist temperature as a complementary sensor to movement-based ones could enable their strengths to be leveraged and fused to support more accurate sleep detection.

More accurate sleep detection for older adults using wearable systems could support better short-term and long-term monitoring of sleep. Through daily sleep tracking, irregular sleep quality and patterns can be recognized by machine learning algorithms and then be reported to older adults or their health care providers. Such monitoring systems are especially useful for OAWD because the incidence of poor sleep is quite high in this population, and self-reporting of sleep is often difficult or impossible. Wearable technologies for sleep tracking and sleep problem identification could help to better understand older adults’ sleep using objective measures, but this approach is only possible if the results are accurate. Accurate sleep data would enable the creation of tailored sleep management plans, enabling better sleep support for each individual, thus supporting better health outcomes and quality of life.

### Limitations

This study had several limitations. First, only the Mi Band 2 was the only commercially available wearable device that was examined. As other commercially available smart wristbands collect other types of data (eg, PPG) and use different sleep detection algorithms, their performance will likely be different. These alternatives should be examined in healthy older adults and OAWD in future studies. Second, the sample size of this study was small. Future research with larger sample sizes is required to provide a deeper, comprehensive understanding that needs to support greater generalization of the results. Third, all our OAWD participants lived in the same LTC home. Data from other LTC homes as well as OAWD living in the community need to be investigated to determine how the environment plays a role in sleep detection and dementia. Finally, sleep onset and offset were estimated from sleep journals and/or actigraphy data. Using another method, such as computer vision, could estimate onset and offset more accurately as well as provide information about what was happening during sleep (eg, giving insight into repetitive movements).

### Conclusions

This research adds evidence that wrist temperature can be used as an indicator of sleep status, including for OAWD and people with irregular sleep patterns. As illustrated by case studies from our data, suboptimal sleep detection performance by a commercial wristband was likely because of broken sleep patterns and body movements. As wearable technologies are increasingly being used by LTC homes and healthy older adults to track sleep and inform sleep management and support, this research suggests that caution should be used when interpreting sleep data when monitoring the sleep of older adults, particularly those living with dementia. This highlights the need for future research and development to create systems that better complement older adult populations. Future sleep monitoring wearable systems could consider adding a temperature sensor to capture the waist temperature as an extra indicator of sleep combined with motion-based data and machine learning algorithms of typical sleep patterns.

## Abbreviations

CBT: core body temperature
ESS: Epworth Sleepiness Scale
IS: interday stability
IV: intraday variability
LTC: long-term care
MMSE: Mini-Mental State Examination
OAWD: older adults living with dementia
PSG: polysomnography
PSQI: Pittsburgh Sleep Quality Index
PSW: personal support worker
